# Plant Extract-Based Fabrication of Silver Nanoparticles and Their Effective Role in Antibacterial, Anticancer, and Water Treatment Applications

**DOI:** 10.3390/plants12122337

**Published:** 2023-06-15

**Authors:** Umar Farooq, Ahmad Kaleem Qureshi, Hadia Noor, Muhammad Farhan, Mohammad Ehtisham Khan, Osama A. Hamed, Abdullateef H. Bashiri, Waleed Zakri

**Affiliations:** 1 Department of Chemistry, The Islamia University of Bahawalpur, Baghdad-ul-Jadeed Campus, Bahawalpur 63100, Pakistan; umarfarooqe2@gmail.com; 2 Department of Chemistry, University of Sahiwal, Sahiwal 57000, Pakistan; akq999@yahoo.com; 3 Centre of Excellence in Solids State Physics, University of the Punjab, Quaid Azam Campus, Lahore 54590, Pakistan; 4 Department of Chemical Engineering Technology, College of Applied Industrial Technology, Jazan University, Jazan 45142, Saudi Arabia; 5 Department of Mechanical Engineering Technology, College of Applied Industrial Technology, Jazan University, Jazan 45142, Saudi Arabia; 6 Department of Mechanical Engineering, College of Engineering, Jazan University, Jazan 45142, Saudi Arabia; abashiri@jazanu.edu.sa (A.H.B.); wzakri@jazanu.edu.sa (W.Z.)

**Keywords:** silver nanoparticles, *Ammi visnaga*, antibiofilm, anticancer, Eosin Y, water treatment

## Abstract

*Ammi visnaga* is a biennial or annual herbaceous plant belonging to the family *Apiaceae*. For the first time, silver nanoparticles were synthesized using an extract of this plant. Biofilms are a rich source of many pathogenic organisms and, thus, can be the genesis of various disease outbreaks. In addition, the treatment of cancer is still a critical drawback for mankind. The primary purpose of this research work was to comparatively analyze antibiofilms against *Staphylococcus aureus*, photocatalytic activity against Eosin Y, and in vitro anticancer activity against the HeLa cell line of silver nanoparticles and *Ammi visnaga* plant extract. The systematic characterization of synthesized nanoparticles was carried out using UV–Visible spectroscopy (UV-Vis), scanning electron microscopy (SEM), energy-dispersive X-ray spectroscopy (EDX), atomic force microscopy (AFM), dynamic light scattering (DLS), zeta potential, and X-ray diffraction microscopy (XRD). The initial characterization was performed with UV-Vis spectroscopy, where a peak appeared at 435 nm, which indicated the SPR band of the silver nanoparticles. AFM and SEM were performed to determine the morphology and shape of the nanoparticles, while EDX confirmed the presence of Ag in the spectra. The crystalline character of the silver nanoparticles was concluded with XRD. The synthesized nanoparticles were then subjected to biological activities. The antibacterial activity was evaluated by determining the inhibition of the initial biofilm formation with *Staphylococcus aureus* using a crystal violet assay. The response of the AgNPs against cellular growth and biofilm formation was found to be dose dependent. Green-synthesized nanoparticles showed 99% inhibition against biofilm and bacteria, performed excellent anticancer assay with an IC_50_ concentration of 17.1 ± 0.6 µg/mL and 100% inhibition, and photodegradation of the toxic organic dye Eosin Y up to 50%. Moreover, the effect of the pH and dosage of the photocatalyst was also measured to optimize the reaction conditions and maximum photocatalytic potential. Therefore, synthesized silver nanoparticles can be used in the treatment of wastewater contaminated with toxic dyes, pathogenic biofilms, and the treatment of cancer cell lines.

## 1. Introduction

Cancer is one of the primary causes of death in the whole world [1], while bacteria-related diseases stand in second place [2,3]. Some genres of cancer are reputed to endure mutation. This alteration in cancer cells results in the spread of cancer to other organs, sometimes empowering the cancer to recur. Current medications that are known to control/hinder the growth of cancer cells also produce serious side effects in patients, resulting in a reduction in the quality of life [4,5]. Over the years, this outcome has continued, as unfortunately no drug has been discovered that can provide maximum results with minimal side effects [6,7,8]. 

In humans, *Staphylococcus aureus* is habitually resident in the skin and nasopharynx. It can originate in different infections, including in soft tissue, internal organs, and endovascular sites. Food contaminated with *Staphylococcus aureus* can result in food poisoning. Biofilms are found to be highly resistant to traditional authentic treatments, functioning as reservoirs for various classes of pathogenic microorganisms; this results in making microorganisms residing in these biofilms more impervious to treatment. Bacterial biofilms are commonly preserved inside a self-made extracellular polymeric substance (EPS) that contains lipids, deoxyribonucleic acid (DNA), and exopolysaccharide [9,10]. At this stage, the EPS matrix hinders the penetration of traditional antibiotics against particular bacteria, making it up to 1000-fold resistant to several classes of antibiotic drugs by neutralizing them using their extracellular polysaccharides [10,11]. Therefore, a broad spectrum of antibiotics are found to be ineffective in destroying biofilm cells and stationary phase cells, which rely on nominal nutrition to survive [12]. Thus, there is an urgent need to design extensive antibiotic drugs, which might be either synergetic or additive [13]. 

Over the years, silver nanoparticles have become well known for their superior antibacterial activity against a vast class of bacteria, including *Pseudomonas aeruginosa, Escherichia coli*, and *Staphylococcus aureus* [14,15,16,17,18,19,20,21]. However, the mechanism of its bioactivity is still poorly understood. Some reports have revealed that the mode of action of silver nanoparticles is the same as that of silver ions [22]; however, the proposed mechanism of its action can be outlined as AgNP deposits on bacterial cell walls that deactivate the essential enzymes present inside the cells [23], followed by the production of reactive oxygen products, including hydroxyl radicals, hydrogen peroxide, and superoxide anion [13,24].

The purpose of this study was to synthesize plant-mediated silver nanoparticles stable enough to work as strong antibacterial agents against a vast class of bacteria. Furthermore, in terms of curing cancer, they must be less cytotoxic, possessing suitable anticancer activity so that we can stand a chance of designing anticancer medicines. As all metallic nanoparticles are found to be cytotoxic, there is still the possibility of mediating them with different drug derivatives that are not cytotoxic and not as active towards cancer cell lines; hence, delivering those drug derivatives with silver nanoparticles could be a potential avenue. In addition, the synthesized silver nanoparticles could work as catalysts in water-treatment applications [25,26]. Overall, the multifaceted application of synthesized silver nanoparticles via a green route could be utilized in several varied applications.

### Rationales for the Activities Performed

The rationales for the three activities performed in the present research work are as follows:

- Cytotoxicity assay: The rationale for performing the cytotoxicity assay was to evaluate the potential of the green-synthesized silver nanoparticles (AgNPs) as an anticancer agent. The assay was performed on two different cell lines, MCF-7 and MDA-MB-231, to determine the toxicity of AgNPs towards breast cancer cells. The results of the assay were compared with the standard drug Idarubicin to assess the efficacy of AgNPs as a potential anticancer agent.
- Photocatalytic activity against Eosin Y: The rationale for performing the photocatalytic activity against Eosin Y was to evaluate the potential of AgNPs as a photocatalyst for the degradation of the Eosin Y dye. Eosin Y is a widely used water-soluble dye in the textile and paper industries, and its metabolites are carcinogenic to both human and aquatic ecosystems if disposed of untreated. The degradation of Eosin Y was performed in the presence of NaBH_4_ to better evaluate the role and the competence of green-synthesized AgNPs.
- Antibiofilm activity: The rationale for performing the antibiofilm activity was to evaluate the potential of AgNPs as an antibiofilm agent. Biofilm formation is responsible for about 65% of all microbial and 80% of acute infections. Microbial cells in biofilms possess 10–1000 times more antibiotic resistance in contrast with planktonic cells. Therefore, hindering the biofilms at the early stage of attachment may result in being a key factor in finding promising antibiofilm agents. The effect of AgNPs on the inhibition of initial biofilm formation by Staphylococcus aureus was determined by crystal violet assay.

## 2. Experimental Section

### 2.1. Ammi visnaga Plant

**Figure 1:**
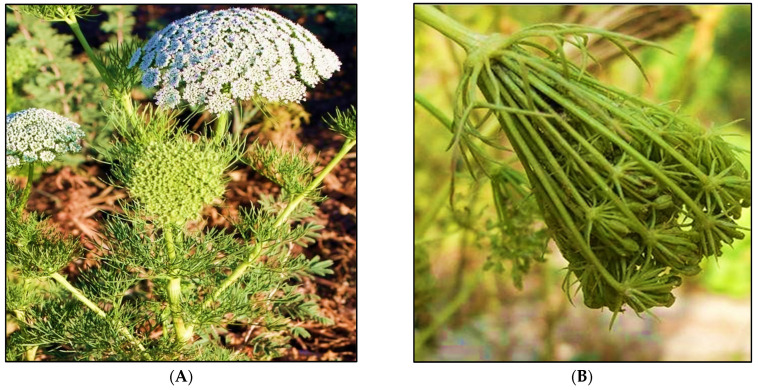
*Ammi visnaga* flower (**A**) and stem (**B**).

*Ammi visnaga* is a biennial or annual herbaceous plant and belongs to the family *Apiaceae*. It is a herb, and its leaves are approximately 20 cm in length, and oval triangular in shape. Its stem is usually erect, cylindrical, furrowed, highly branched, and entirely covered with leaves. The flowering on this plant occurs around June and provides tetracyclic, pentamerous, white flowers having a radial symmetry. The inferior ovary is comprised of two united carpels and its root is fattened, resembling that of carrot root. The fruit is a compacted oval-shaped structure, possessing two mericarps and is approximately 3 mm in length. *Ammi visnaga* is also known as bisnaga, toothpick plant, khella, toothpick *ammi*, and Bishop’s weed. This plant grows in the entire world and is indigenous to Western Asia (Cyprus, Syria, Turkey, Iran, Lebanon, Iraq, and Israel), Northern Africa (Morocco, Tunisia, Algeria, and Libya), Southeastern Europe (Greece, Albania, and Italy), Southwestern Europe (France, Spain, and Portugal), and the Caucasus region (Armenia, Georgia, and Azerbaijan) (Figure 1). 

### 2.2. Important Chemical Constituents Reported from Ammi visnaga

To better evaluate the role of chemical constituents in the synthesis and stabilization of nanoparticles, a review of the literature was carried out. Some of the important reported chemical constituents were *Khellin*, *visnagin, khellinol*, *ammiol*, *khellol* [27], *visnadine* [27,28], *samidin* [28], *dihydrosamidin* [29], bornyl acetate, croweacin, 2,2-dimethylbutanoic acid, isobutyl isobutyrate, thymol, linalool [30], (E)-ß-ocimene, ɑ-terpinene, transthujene, cis-pinene hydrate, linalool, methyl octadecanoate, isoamyl-2-methyl butyrate, isopentyl isovalerate, ɑ-isophorone, thymol, 2,2-dimethyl butanoic acid [31,32,33], quercetin, kaempferol [34], rhamnetin, isorhamnetin, rhamnazin, 3-O-glucoside of rhamnetin, 3-O-glucoside of isorhamnetin, 3-O-glucoside of rhamnazin, 7-O-glucoside of isorhamnetin, 3-O-rutin of quercetin, 3-O-rutin of isorhamnetin, and Quercetin 7, 3, 4′ –O-triglucoside (Table 1) [35].

### 2.3. Materials and Instrumentation

Silver nitrate (AgNO_3_, 99.8%), Eosin Y (C_20_H_6_Br_4_Na_2_O_5_), and ethanol (C_2_H_5_OH, 99%) were bought from Sigma-Aldrich and were subjected to the experiment without any additional purification. All molar solutions were prepared using double distilled water to avoid any possible contamination. Instruments used for the characterization included (Evolution 300-USA) UV–Visible spectrophotometer for UV–Visible spectroscopy, 5500 Atomic Force Microscope (Agilent Technologies, Santa Clara, California, USA) for atomic force microscopy, Bruker D8 venture X-ray powder diffractometer for XRD analysis, Nano ZS zeta sizer system (Malvern instruments-UK) and Apreo 2 C LoV ac (Thermo Fisher Scientific, Waltham, MA, USA) for scanning electron microscopy and energy dispersive spectroscopy. 

### 2.4. Synthesis of Silver Nanoparticles

**Scheme 1:**
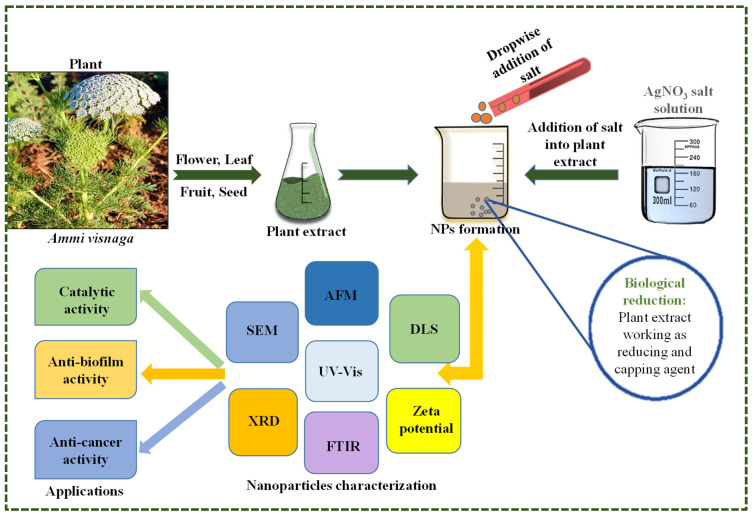
Schematic illustration of reaction mechanism steps.

The glassware selected for the experiment was washed and dried carefully. Chilled distilled water was used to prepare a stock solution of 1 mM silver nitrate to avoid the decomposition of salt. A conical flask carrying 50 mL of 1 mM AgNO_3_ solution was kept on a hot plate with constant magnetic stirring; then, *Ammi visnaga* extract was poured dropwise into the flask. The stirring continued for 2 h at room temperature until a color change from light yellow to blackish brown was observed. Then, the sample was removed from stirring and placed in complete darkness for 24 h for complete reduction of Ag^+1^ ions to Ag^0^. After this, the solution was parted through centrifugation for 15 min at 6000 RPM, washed with ethanol to remove any remaining impurities, and kept in the refrigerator at 4 °C for further characterization and applications (Scheme 1).

### 2.5. Methodology for Antibiofilm Activity

#### 2.5.1. Bacterial Strain and Medium

*Staphylococcus aureus* (ATCC 6538) is a strong biofilm-forming strain [4]. Brain heart infusion (BHI) broth was used to prepare frozen cultures, which were reinforced with a 10% (vol/vol) solution of glycerol and kept at −80 °C for likewise experiments. These frozen cultures were defrosted before the experiment plan, spread on Mannitol Salt Agar (MSA) plates by a sterile wire loop, and processed for incubation at 37 °C for a day. From these subcultures, cells were taken from one colony of the preceding culture and sustained weekly on mannitol salt agar plates.

#### 2.5.2. Crystal Violet Assay for Antibiofilm Activity

Antibiofilm activity of AgNPs was performed in sterile flat-bottom 96-well polystyrene microtiter plates as described by [36]. Inoculation of bacterial cells was carried out in brain–heart infusion (BHI) broth provided with 0.25% solution of glucose (BHIg) and cultivated at 37 °C for 18–24 h. These fully grown cells were diluted 1:1000 into fresh BHIg and 100 μL of its quantity was introduced to the wells accommodating different concentrations of AgNPs with BHIg to achieve a total volume of 200 μL per well. Wells, having BHIg with bacterial cells, were selected as the positive control, and only BHIg-containing wells were selected as the negative control. Incubation of the plates was performed at 37 °C for 24 h. After incubation, the planktonic cell growth was calculated by evaluating the OD at 600 nm with a Multiskan™ GO spectrophotometer (Thermo Fisher Scientific, USA) before dispensing the medium. The plate was then washed multiple times with double-distilled water and retained in a hot air oven at 60 °C for an hour. Staining of the wells was completed with 200 μL of 0.1% crystal violet solution for 15–20 min. Each well was then washed again with distilled water and well-dried before adding 200 μL of glacial acetic acid (30% *v*/*v*) to solubilize the biofilm stain. Finally, the measurement of biofilm production was taken corresponding to the OD at 595 nm. Measurements were taken in replicas of three, and the assay was rerun three times. The percent inhibition of either planktonic cells growth or biofilm was assessed using this formula [36]

% Inhibition = 100 − [(ƛ Treated − ƛ Blank)/ (ƛ Untreated − ƛ Blank) × 100]

### 2.6. Anticancer Activity Protocol

Cytotoxic assay and anticancer activity of prepared silver nanoparticles were performed in 96-well flat-bottomed microplates employing the standard MTT (3-[4, 5-dimethylthiazole-2-yl]-2, 5-diphenyl-tetrazolium bromide) colorimetric assay. To proceed with the experiment, HeLa cells (cervical cancer) were cultivated in Minimum Essential Medium Eagle, provided with 5% of fetal bovine serum (FBS), 100 IU/mL of penicillin, and 100 µg/mL of streptomycin in 75 cm^2^ flasks, and put in 5% CO_2_ incubator at 37 °C. Exponentially developing cells were cultured, counted on a hemocytometer, and diluted with a certain medium. A selective concentration of cell culture (6 × 10^4^ cells/mL) was prepared and established (100 µL/well) into 96-well plates. The medium was withdrawn after a night of incubation, and a fresh medium of 200 µL was added with distinct concentrations of compounds (1–30 µM). After 2 days, the addition of 200 µL MTT (0.5 mg/mL) was accomplished and carried out for incubation for an additional 4 hrs. In due course, 100 µL of DMSO was also instilled to each well. The rate of MTT reduction against formazan in the cells was calculated with the help of a microplate reader (Spectra Max Plus, Molecular Devices, SAN Jose, CA, USA) by evaluating the absorbance at 570 nm. The concentration effect of 50% growth inhibition (IC_50_) was considered to calculate the cytotoxicity. The formula given below was used to calculate the percent inhibition:

% inhibition = 100 − ((mean of O.D of test compound − mean of O.D of negative control)/(mean of O.D of positive control − mean of O.D of negative control) × 100).

The results (% inhibition) were processed using the SoftMax Pro 6.2 GxP software (Molecular Device, UK).

### 2.7. Photocatalytic Activity against Eosin Y

The photocatalytic evaluation for the dye (pure Eosin Y) was purchased from Sigma-Aldrich. The as-synthesized silver nanoparticles were evaluated for their catalytic potential. In a customary experiment, a 50 µL solution of pure dye was mixed with 1.5 mL of de-ionized water and its UV-Vis spectra were recorded. Then, a 0.2 mL solution of NaBH_4_ (0.2 M) was added to the same glass cuvette to observe the catalytic action of NaBH_4_. After the addition of NaBH_4_, only a minute fall in the UV-Vis spectrum was noted which was an indication of the lower catalytic capability of NaBH_4_. We waited for half an hour but there was no more alteration in the spectra. Finally, the solution of AgNPs (400 µL of 62.5 µM) was prepared and added into a glass cuvette carrying dye and NaBH_4_ solution. This mixture was then stirred for a few hours to better permit the adsorption of dye molecules on the surface of nanoparticles. After the addition of AgNPs, the characteristic absorbance peak of Eosin Y started to fall indicating that nanoparticles have started the catalytic activity. Different time intervals spectra were obtained until there was no more observance of the spectrum fall. Two factors, pH and dosage of photocatalyst, were examined which resulted in an increase in both pH and dosage of photocatalyst causing an increase in catalytic activity. The whole experiment was performed at room temperature and stirring continued throughout the whole demonstration. The degradation rate was calculated with the help of the following formula:

Degradation rate (%) = (C_o_ − C_t_/C_o_) × 100 (1)

where C_o_ is the initial absorbance value while C_t_ is the final absorbance value taken before and after the experiment. The chemical kinetics of the reaction can be acquired by the given formula:

−Ln(C/C_o_) = kt (2)

where k is the rate constant; C_o_ is the initial dye concentration; and C is the final concentration of dye at given time t.

## 3. Results

### 3.1. UV-Vis Spectroscopy

**Figure 2:**
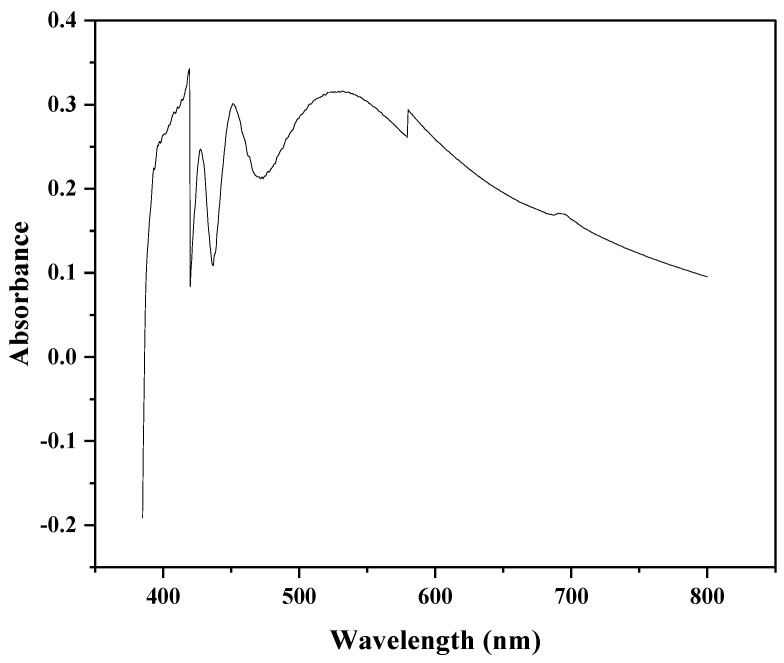
UV-Vis spectra of pure plant crude of *Ammi visnaga*.

UV-Vis spectroscopy is the most prominent and indispensable technique in the characterization of nanoparticles. Figure 2 displayed the UV-Vis spectra of pure plant crude. The characteristic absorbance peak of *Ammi visnaga* was observed at 538 nm.

**Figure 3:**
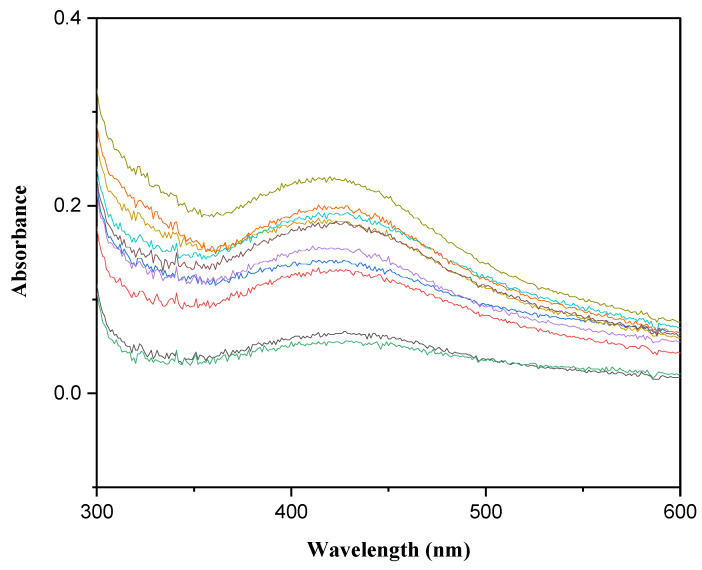
UV-vis spectral readings taken from the start of the synthesis reaction of AgNPs until completion.

As soon as the synthesis reaction of AgNPs started with mixing the salt with the plant extract, the color of the solution turned light yellow and ultimately dark brown. At this moment, the solution was subjected to UV-Vis spectroscopy. The absorbance was set from 0 to 2 and a characteristic peak of Ag at 435 nm was observed (Figure 3).

### 3.2. FTIR Analysis of Prepared AgNPs

The FTIR study was carried out to evaluate the role of important chemical constituents responsible for the synthesis and stabilization of nanoparticles. FTIR is basically a clear identification of phenolic, phenolic acids, and alkaloidal functional groups that work as capping with AgNPs resulting in the formation of nano silver. Spectrum recorded gave multiple peaks directing the complexity of biological matter [37].

**Figure 4:**
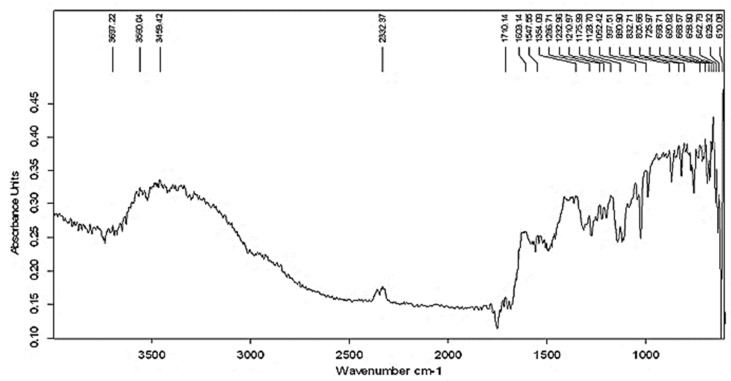
FTIR spectrum of prepared silver nanoparticles.

The FTIR analysis display various stretches of different bands at 3459.42, broad (hydroxyl group, OH, H-bonded), 2332.37 (OH-stretched), 1710.14 (carboxylic acid), 1609.14 (primary amides), 1547.55 (alcohols), 1354.09 (ethers C-O), 1296.71–1210.97 (esters), 1175.99–1128.70, 1052.42, 997.51, 890.90–806.66, 725.97, and 698.71–610.08 cm^−1^ (halogen functionalities, series of bands indicating the presence of fluorine, two or more bands are indicating C-Cl and C-Br stretch). Chemical constituents reported from *Ammi visnaga* can be illustrated by the presence of these functional groups (Figure 4).

### 3.3. Dynamic Light Scattering and Zeta Potential

**Figure 5:**
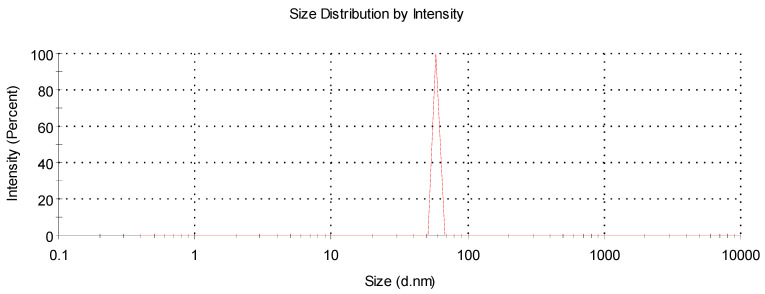
DLS image of synthesized silver nanoparticles.

**Figure 6:**
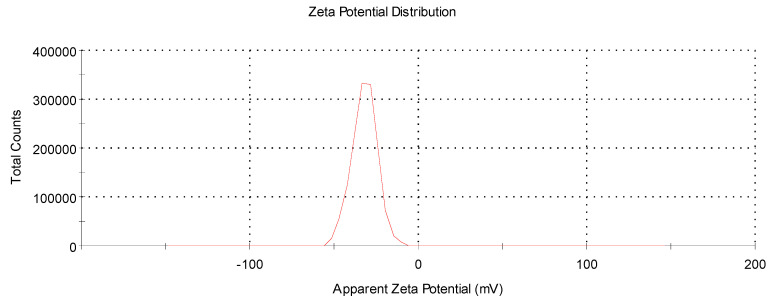
Zeta potential image depicting the surface charge of silver nanoparticles.

To ascertain the surface charge and particle size, DLS and zeta potential were performed [38]. While zeta potential is also known as *electrokinetic potential*, it can be utilized for the elucidation of colloid stability. Zeta potential values of ±30 mV, ±20–30 mV, ±10–20 mV, and ±0–10 mV can be narrated as highly stable, moderately stable, stable, and highly unstable, respectively. DLS spectra in Figure 5 showed that the average size of nanoparticles observed is 58.77 nm while the zeta potential image given in Figure 6 illustrated that a surface charge of −31.9 mV can be hinted as nanoparticles present in solution are highly stable. Moreover, the observed standard deviation value in DLS was 22.16 (d.nm), while in the case of the zeta potential the recognized standard deviation was 7.76 mV.

### 3.4. Atomic Force Microscopy

**Figure 7:**
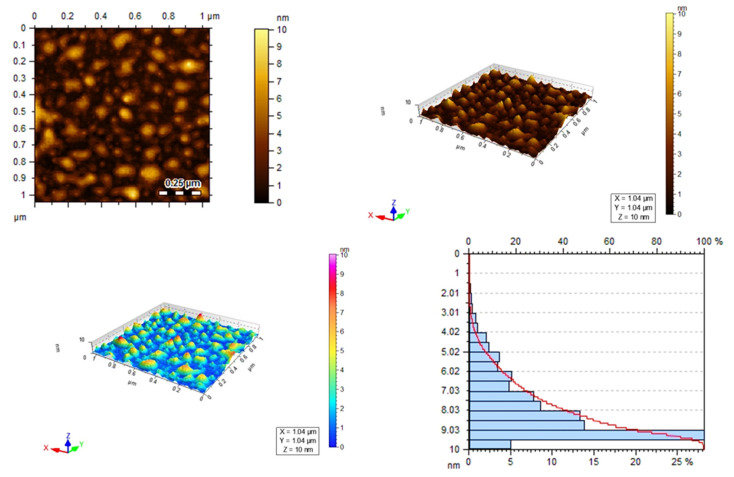
AFM images of synthesized silver nanoparticles.

The atomic force microscopy is utilized for its capability to harvest the images of high resolution (typically ~0.2 nm to 1 nm). Some quantity of sample solution was taken and subjected for analysis. The topographical 3D images of the prepared silver nanoparticles are given below. It can be ascertained that the calculated size of AgNPs from AFM analysis after determining the height profile, evaluating the dimensions of AgNPs, and comparing statistical analysis is approximately 6.018 nm (Figure 7).

### 3.5. Scanning Electron Microscopy and Energy Dispersive X-ray Spectroscopy

**Figure 8:**
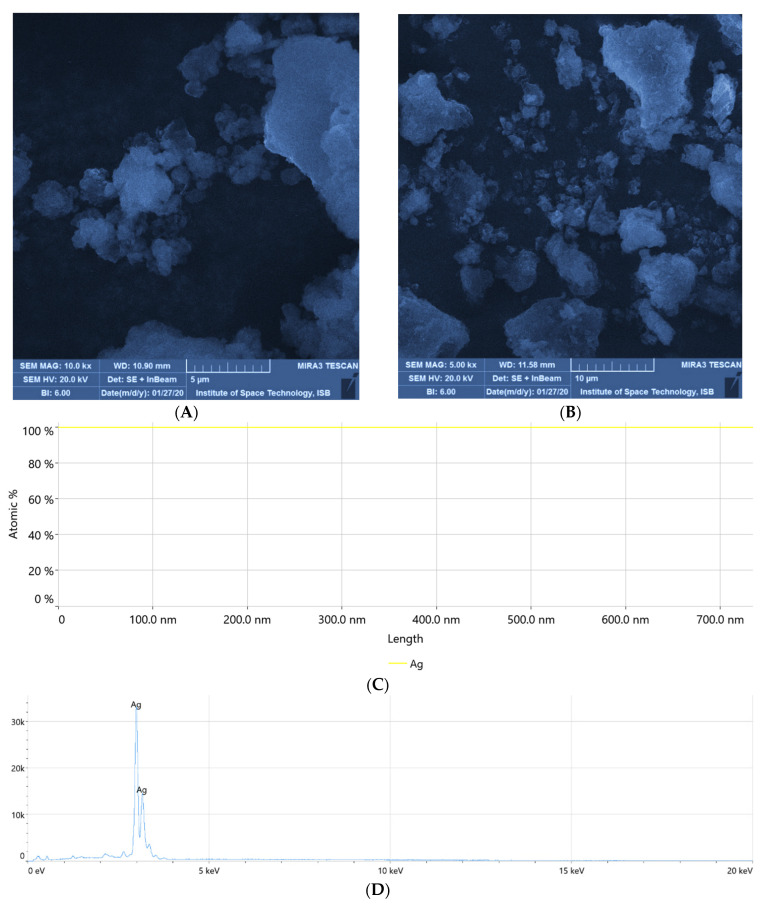
(**A**,**B**) SEM images showing structural morphology (**C**,**D**) EDX images display atomic weight % and the chemical composition of prepared silver nanoparticles.

SEM along with EDS was performed to determine the surface morphology along with biological components of synthesized silver nanoparticles. Scanning electron microscopy is a marvelous tool in order to determine the hidden complexity and details of nanoparticles which are beyond the reach of light microscopy [39]. Meanwhile, EDS was performed to verify the presence of silver and other chemical constituents. The dried sample (slightly heated on a hotplate) was carefully ground to avoid the visibility of cluster formation. The SEM and EDS images observed are given in Figure 8. It can be observed in the pictures that some of the nanoparticles formed clusters that can be attributed to the fact that “AgNPs tend to have a high surface energy, leading to a tendency to aggregate or cluster together. This presence of clusters in SEM images could be attributed to these interparticle interactions, where neighboring nanoparticles come into contact and form aggregates”. Some well dispersed nanoparticles can also be observed through the surface of the cell and are round. Moreover, the presence of Ag peaks can also be examined from the EDS images.

### 3.6. X-ray Diffraction

**Figure 9:**
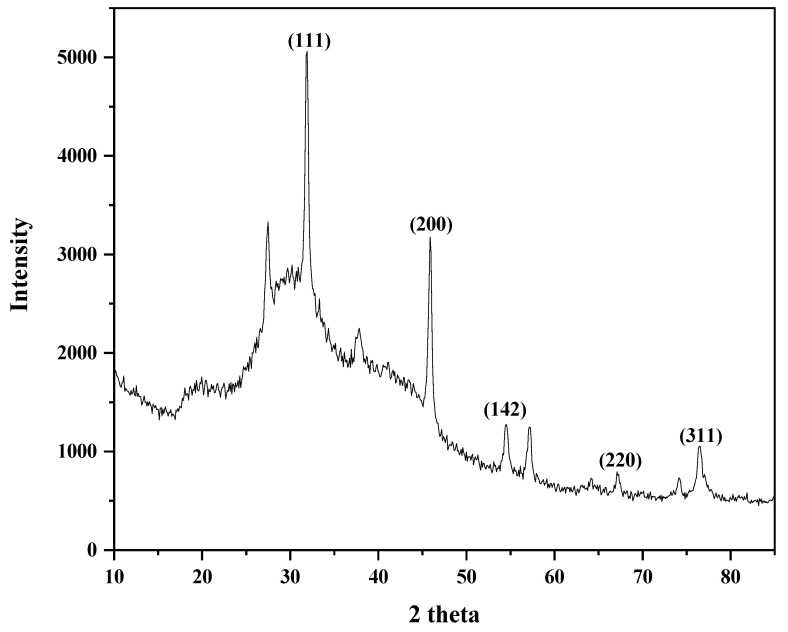
XRD spectra denoting the crystalline nature of silver nanoparticles.

Powder XRD was performed to demonstrate the crystalline nature, shape, and lattice parameters of silver nanoparticles. Figure 9 illustrates the diffraction pattern acquired from nanoparticles. Peaks were observed at 2θ values of 31.707, 45.676, 54.419, 67.081, and 76.327 degrees corresponding to (111), (200), (142), (220), and (311) planes of silver. Results obtained were then matched with the standard powder diffraction card of JCPDS, silver file No. 04-0783 [40]. Some minor peaks appearing cannot be comprehended which might be a result of reagents that were left unreacted throughout the reaction.

### 3.7. Antibiofilm Activity

#### 3.7.1. Planktonic Cells and Biofilm Inhibition

A scientific report by the National Institutes of Health suggests that biofilm formation is responsible for about 65% of all microbial and 80% of acute infections [41]. Microbial cells in the biofilms possess 10–1000 times more antibiotic resistance in contrast with the planktonic cells [42]. It is well known that during the preliminary stage of disease evolvement, planktonic cells always tend to become attached to the substratum or the surface available for colonization. This results in the shaping and formulation of biofilms. Therefore, hindering the biofilms at this early level of attachment may result in being a key factor in finding promising antibiofilm agents [24]. The effect of AgNPs on the inhibition of initial biofilm formation by *Staphylococcus aureus* was determined by crystal violet assay. The response of AgNPs against cellular growth and biofilm formation is dose dependent. At higher concentrations, both the antibacterial and antibiofilm activities are high and decrease gradually at lower concentrations.

**Figure 10:**
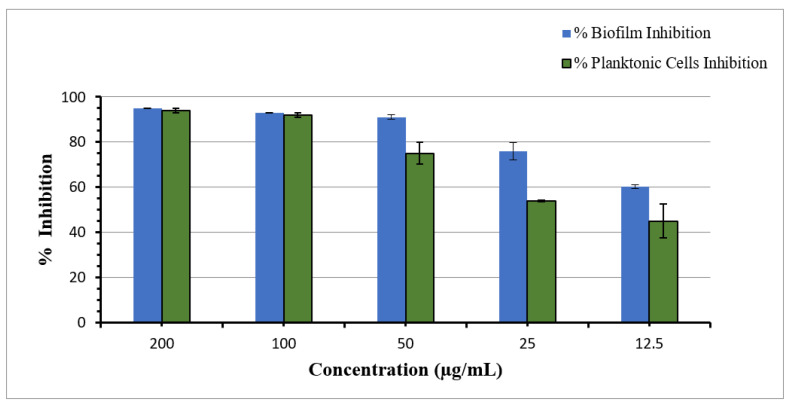
The inhibition of biofilm with different concentrations of silver nanoparticles.

The effect of AgNPs on cellular growth and biofilm is shown in Figure 10 below.

#### 3.7.2. Light Microscopy

**Figure 11:**
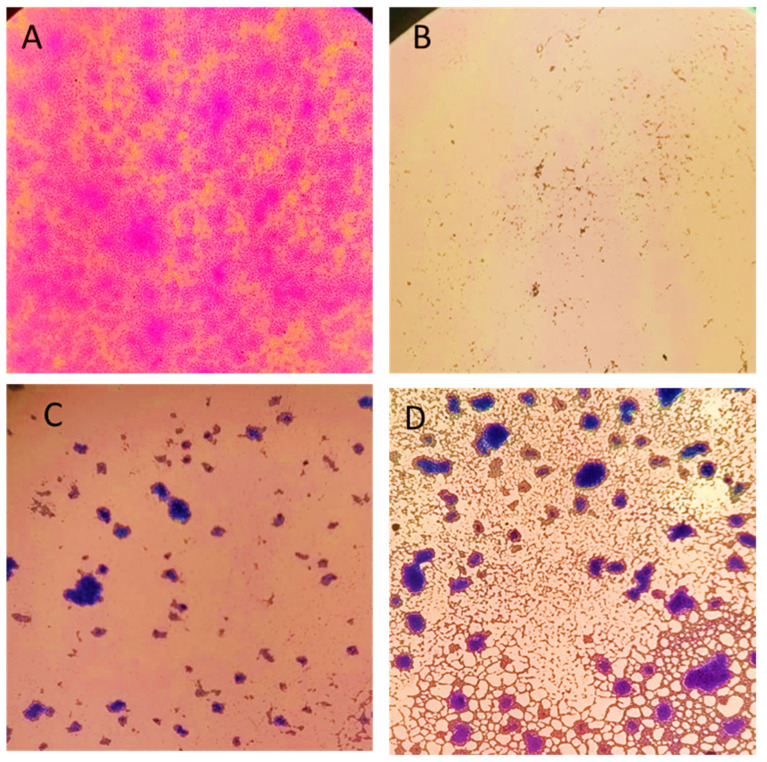
Microscopic images of *Staphylococcus aureus* (ATCC 6538) biofilm inhibition. Scan area in (**A**) is untreated control, while (**B**–**D**) is the result of treating AgNPs at 50 µg/mL, 25 µg/mL, and 12.5 µg/mL, respectively.

Light microscopic analysis of the biofilms formed in the presence and absence of AgNPs confirmed the inhibition of biofilm formation at different concentrations. Visible reductions in biofilms in the treated and untreated wells were observed after crystal violet staining and the images were taken with digital cameras. Results are shown in Figure 11 below.

### 3.8. In Vitro Anticancer Activity

The synthesized silver nanoparticles were also evaluated for anticancer activity against the HeLa cell line with Idarubicin being used as a standard. In comparison with the standard, the synthesized nanoparticle was found to be weakly active towards the HeLa cell line (IC_50_ = 17.1 ± 0.6 µM) while the parent plant extract was inactive towards cancer cell cytotoxicity. Cytotoxicity of these compounds was also evaluated towards the BJ cell line and the synthesized nanoparticle was found to be weakly active (IC_50_ = 17.91 ± 1.0 µM) while the plant extract showed as inactive towards the normal cell line as compared to standard (IC_50_ = 0.1 ± 0.02 µM). The IC_50_ (half maximal inhibitory concentration) is a quantitative measure used in pharmacology and biochemistry to assess the potency or effectiveness of a compound in inhibiting a specific biological or biochemical function.

### 3.9. Catalytic Activity

**Figure 12:**
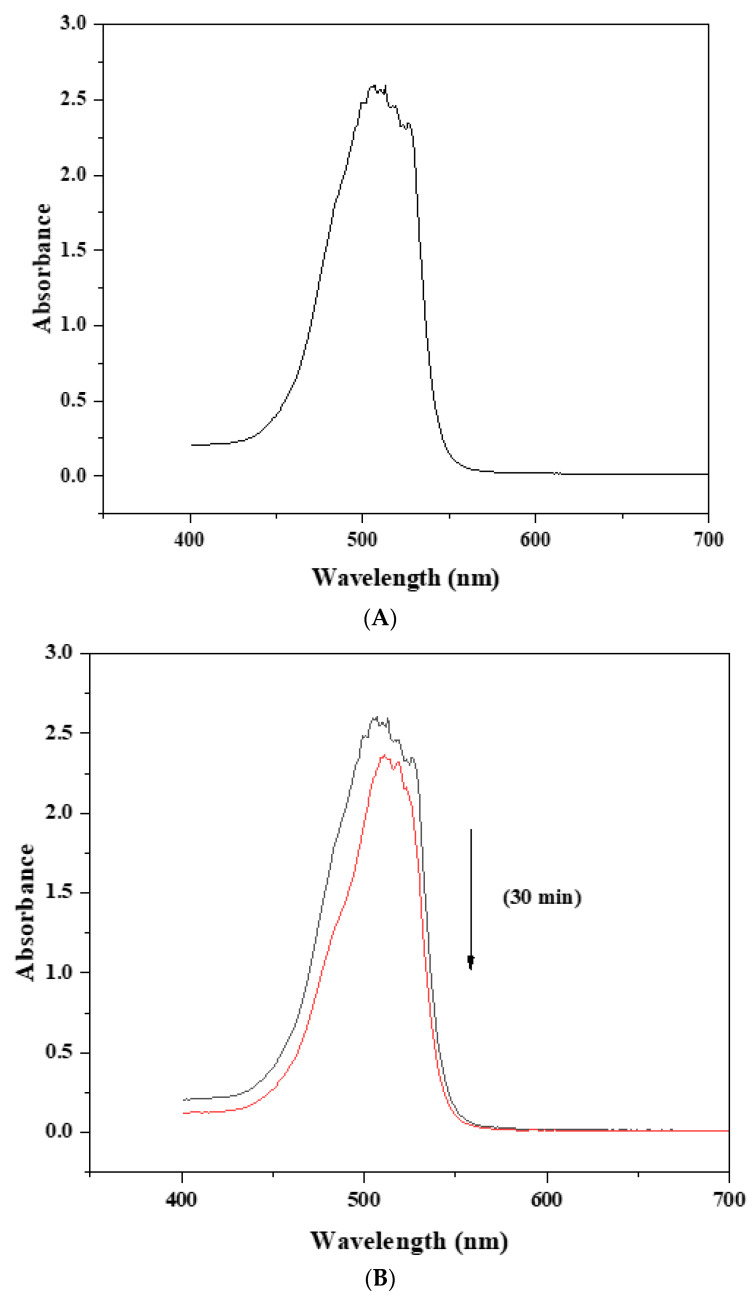

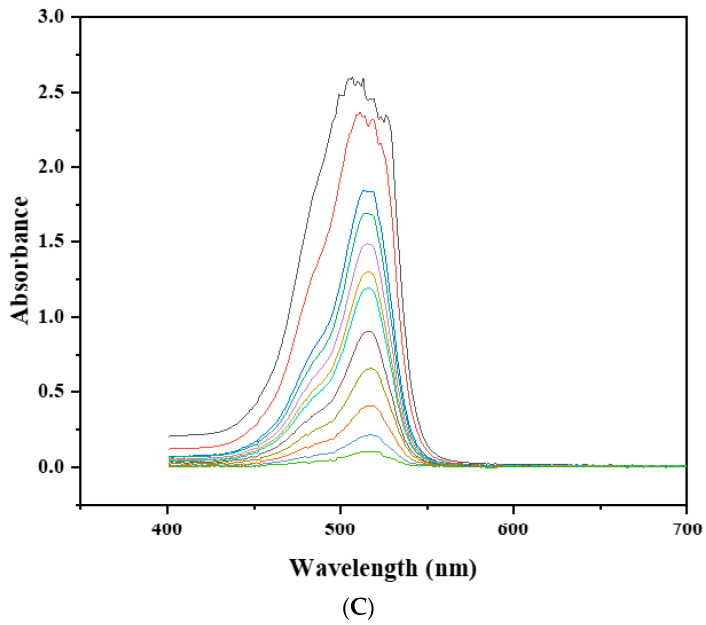
(**A**) UV–Visible spectra of pure Eosin Y dye; (**B**) UV–Visible spectra of Eosin Y dye after treatment with NaBH_4_; (**C**) Time-dependent spectra of Eosin Y after treatment with photocatalyst (AgNPs) at pH 7.0.

Tetrabromofluorescein water soluble dye Eosin Y is widely used in the textile and paper industries [43,44], is a red fluorescent dye, and its metabolites are very carcinogenic to both human and aquatic ecosystems if disposed of untreated [45]. The degradation of Eosin Y was performed in the presence of steep NaBH_4_ to better evaluate the role and the competence of green-synthesized AgNPs. The typical peak of Eosin Y dye was observed at 514 nm in UV-vis spectroscopy [46,47] and can be seen in Figure 12A. After the addition of NaBH_4_, only a minute fall in the peak was noticed which was unaltered for 30 min (Figure 12B). In between, we continued running the UV-vis spectra to ascertain if any other change in the peak happened, but it was the same. So, to proceed with the degradation trend, a dosage of photocatalyst was added. It was noted that as soon as the solution of the photocatalyst was added to the dye solution and irradiation was started, a characteristic fall in the absorbance value was observed hinting that the photocatalyst has started the degradation. Spectra were run after every 2 min and there was no more activity after 20 min (Figure 12C). The rate constant calculated for the degradation was 0.0526 min^−1^. The activity was performed at room temperature at pH 7.0. Moreover, blank tests with no catalyst were also performed which performed zero considerable degradation, hinting that there is no such way to treat Eosin Y in the absence of the catalyst. The percentage degradation calculated was 95% which indicates the high efficacy of the photocatalyst in the degradation process. This suggests that AgNPs may be a promising candidate for the degradation of Eosin Y in various applications, such as wastewater treatment or environmental remediation. It is important to note that the effectiveness of the degradation process may depend on various factors such as the concentration of the photocatalyst, the reaction time, and the pH of the solution. Moreover, the photocatalytic degradation mechanism occurs through the means of an advanced oxidation process taking place on the catalyst’s surface. Upon light irradiation, the valence band’s electrons become excited to the conduction band, resulting in the formation of an electron-hole pair. These holes of the valence band led to the splitting of H_2_O molecules into H^+^. The reported results are consistent and align well with the previously published studies [26,48,49].

#### 3.9.1. Effect of pH

**Figure 13:**
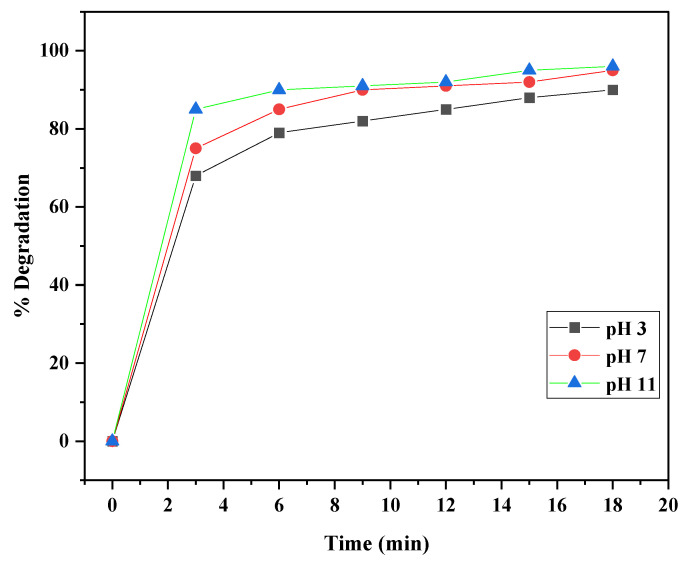
Time profile of percentage degradation of dye with respect to different pH values of photocatalyst (AgNPs).

One of the leading factors that influences the dye degradation reaction is the pH of the solution [50,51,52,53]. The effect of four different pH values, 3, 7, and 11 at a constant value of the photocatalyst was evaluated for the degradation of Eosin Y. The fascinating part was that the degradation potential kept increasing with increasing pH values. This effect, moving from acidic to basic pH values, was also reported in these studies [50,54,55]. The effect of increasing pH on dye’s color removal is well described in Figure 13.

#### 3.9.2. Effect of Photocatalyst Dosage

**Figure 14:**
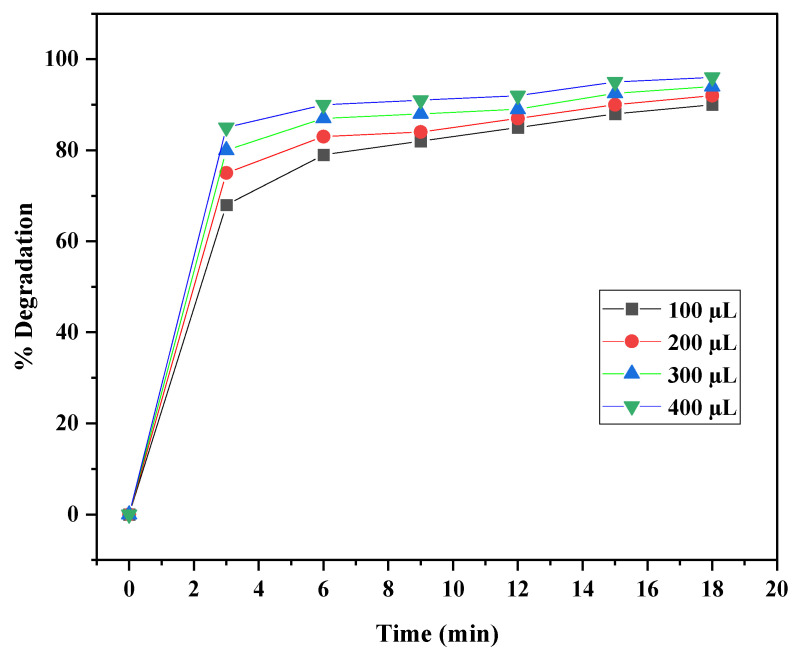
Time dependent % degradation of Eosin Y with different dosages of photocatalyst (AgNPs).

The effect of different photocatalyst dosages (100 µL, 200 µL, 300 µL, and 400 µL) was also analyzed to find out the idea quantity for maximum catalytic potential. It was observed that the maximum quantity (400 µL) of photocatalyst resulted in maximum results. It can be explained by way of the availability of maximum surface area to interact with dye molecules and surface saturation of the catalyst with dye molecules. The same results are also published in these studies [47]. Figure 14 depicts the effect of different dosages of photocatalyst on the degradation of Eosin Y at room temperature.

#### 3.9.3. Reusability of the Photocatalyst

**Figure 15:**
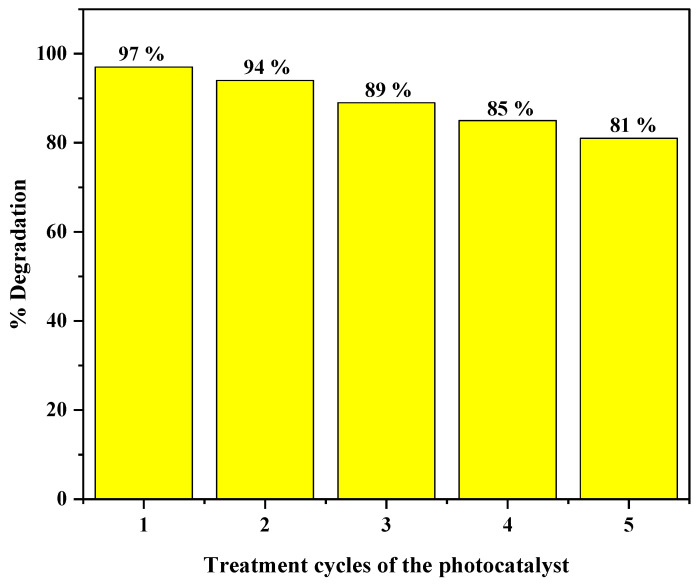
Graphical illustration of treatment cycles of the photocatalyst (AgNPs).

The green-synthesized AgNPs are always favored for the photocatalytic potential of organic dyes as they were much more stable throughout the study. The reusability of the photocatalyst is schematically provided in Figure 15 To recycle the photocatalyst, it was centrifuged from the dye solution, washed with ethanol to extricate any possible contamination, and subjected to further treatments. The fabricated AgNPs nanocatalyst, even after five treatments, displayed peculiar potential. The trend can be explained by demonstrating that after five treatments, the catalytic potential dropped systematically to 97, 94, 89, 85, and 81%, respectively.

#### 3.9.4. Effect of Different Types of Scavengers on Catalytic Performance

**Figure 16:**
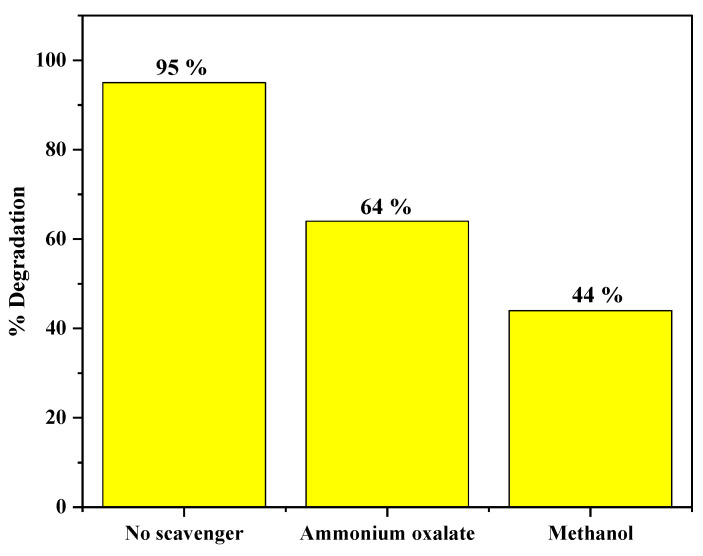
Systematic effect of different scavengers on photocatalytic activity.

Quenching studies were also performed to find out the contribution of reactive intermediates in the photocatalytic potential. Two different types of scavenger’s methanol (OH radical scavenger) and ammonium oxalate (H^+^ scavenger) were used for the experiment. Figure 16 illustrates that the addition of oxalate led to a reasonable loss in degradation resulting in the hindrance of degradation up to 31% (64% degradation occurred). Meanwhile, on the other hand, methanol significantly decreased the degradation by up to 51% (44% degradation occurred).

#### 3.9.5. Mechanism Leading to the Degradation of Products

Upon light irradiation, photocatalytic degradation happens by means of an advanced oxidation process taking place on the catalyst’s surface [56]. The mechanism further continues by the formation of an electron-hole pair caused by the excitation of the valence band’s electrons to the conduction band with the help of irradiation of the photocatalyst by light. These holes of the valence band result in the splitting of the H_2_O molecules into H^+^ and OH^-^. The already shifted electrons of the conduction band, after reacting with oxygen molecules, produce superoxide (O^−2^). This superoxide along with the hydroxyl radical acts as reactive oxygen species (ROS) controlling the degradation mechanism. The hindering effect in the degradation by these ROS is already provided in Section 3.9.4. The integral safe products formed by the degradation of Eosin Y are CO_2_ and H_2_O patterned either by the decomposition of the dye molecules initiated by the OH radical or by the splitting of the C-H and C=C bonds of the dye [57,58].

**Figure 17:**
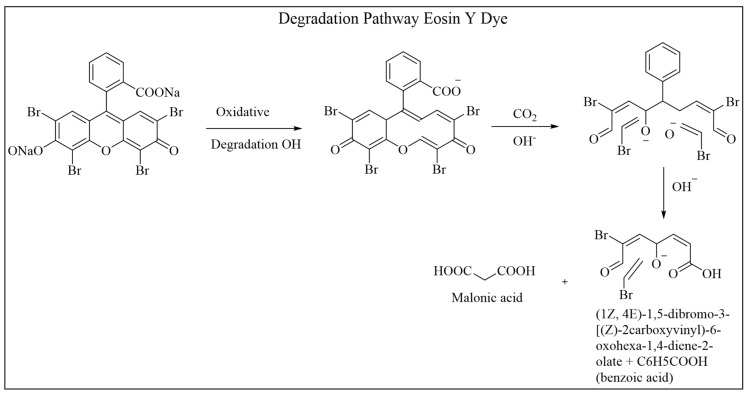
Degradation pathway for photocatalytic degradation of Eosin Y.

The photodegradation process usually initiates with oxidation, by which the whole polycyclic aromatic ring of the dye molecule breakdowns into relatively smaller nontoxic compounds, i.e., carbon dioxide, nitrate, bromide, water, and sulfide. Meanwhile, sometimes the synthesis of organic acids such as oxalic acid and acetic acid is also accompanied [59,60]. Figure 17 depicts the schematic illustration of the degradation route.

## 4. Conclusions

In conclusion, this study demonstrates the potential of green-synthesized AgNPs in anticancer, antibiofilm, and wastewater treatment. The antibiofilm activity of AgNPs was evaluated against *Staphylococcus aureus*, and the results showed that the response of AgNPs against cellular growth and biofilm formation is dose dependent. At higher concentrations, both the antibacterial and antibiofilm activities are high and decrease gradually at lower concentrations. The high efficacy of the photocatalyst was demonstrated through the significant degradation of the dye, with a calculated degradation rate of 0.0526 min^−1^ and a percentage degradation of 95%. The study also highlights the importance of various factors such as the concentration of photocatalyst, the reaction time, and the pH of the solution in determining the effectiveness of the degradation process. Furthermore, the study provides valuable insights into the chemical constituents of *Ammi visnaga*, which can be used to synthesize AgNPs. The characterization techniques used in this study, including UV-Vis spectroscopy, FTIR, DLS, and zeta potential, provide a comprehensive understanding of the properties of the synthesized AgNPs. Overall, the results of this study suggest that green-synthesized AgNPs have great potential for use in various applications, such as wastewater treatment and environmental remediation. Further research is needed to explore these nanoparticles’ full potential and optimize their use in different settings. The findings of this study contribute to the growing body of knowledge on the use of green-synthesized nanoparticles for environmental applications and provide a promising avenue for future research in this field.

## Figures and Tables

**Table 1:** Names of chemical constituents and their structures from *Ammi visnaga*.

S. No.	Compound Name	Structures
**1**	Khellin	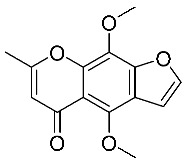
**2**	Visnagin	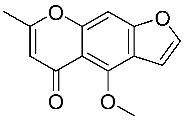
**3**	Khellinol	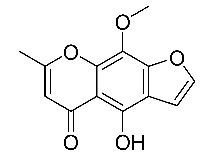
**4**	Ammiol	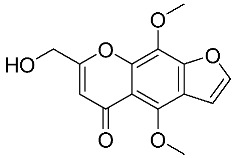
**5**	Khellol	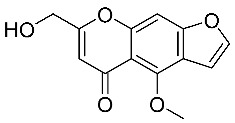
**6**	Visnadine	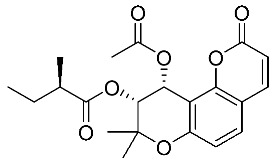
**7**	Samidin	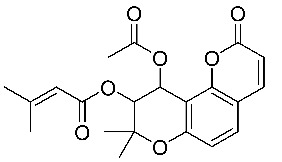
**8**	Dihydro Samidin	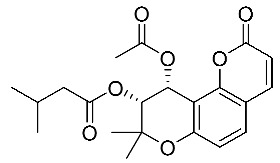
**9**	Bornyl acetate	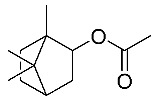
**10**	Croweacin	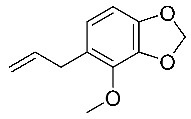
**11**	2,2 dimethyl butanoic acid	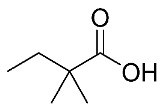
**12**	Isobutyl isobutyrate	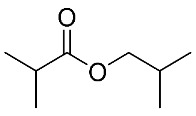
**13**	Thymol	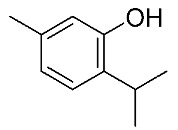
**14**	Linalool	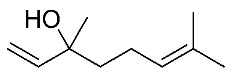
**15**	(E)-β-Ocimene	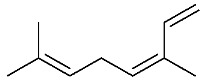
**16**	α-Terpinene	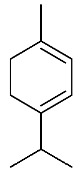
**17**	Trans thujene	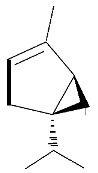
**18**	Cis-pinene hydrate	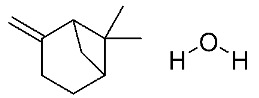
**19**	Linalool	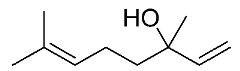
**20**	Methyl octadecanoate	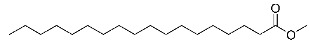
**21**	Isoamyl-2-methyl butyrate	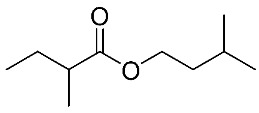
**22**	Isopentyl isovalerate	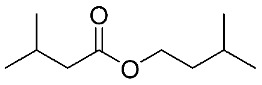
**23**	ɑ-isophorone	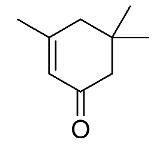
**24**	Quercetin	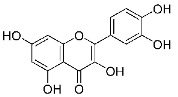
**25**	Kaempferol	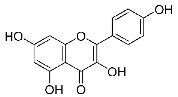
**26**	Rhamnetin	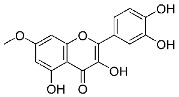
**27**	Isorhamnetin	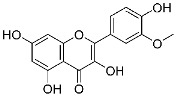
**28**	Rhamnazin	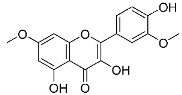
**29**	3-O-glucoside of rhamnetin	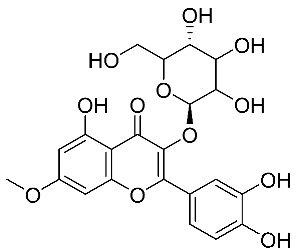
**30**	3-O-glucoside of isorhamnetin	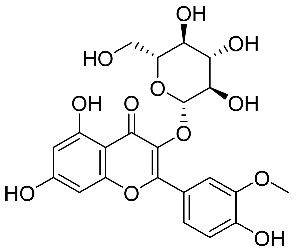
**31**	3-O-glucoside of rhamnazin	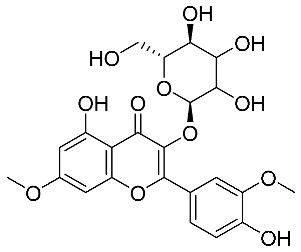
**32**	7-O-glucoside of isorhamnetin	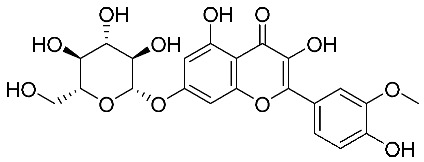
**33**	3-O-rutin of quercetin	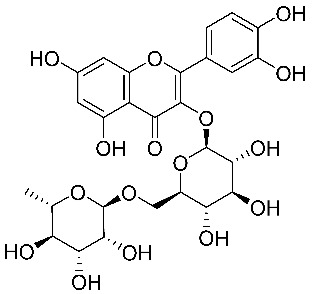
**34**	3-O-rutin of isorhamnetin	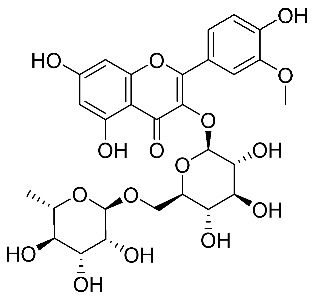
**35**	Quercetin 7, 3, 4′ –O-triglucoside	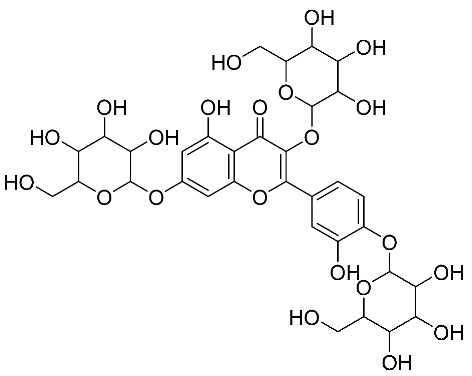

## Data Availability

The data will available upon request.
